# Correlation between clinical risk factors and tracheal intubation difficulty in infants with Pierre-Robin syndrome: a retrospective study

**DOI:** 10.1186/s12871-020-00997-w

**Published:** 2020-04-08

**Authors:** Yanli Liu, Jiashuo Wang, Shan Zhong

**Affiliations:** 1 Science and technology department, China Pharmaceutical University, Nanjing, People’s Republic of China; 2 Research Center of Biostatistics and Computational Pharmacy, China Pharmaceutical University, Nanjing, People’s Republic of China; 3 Department of Anesthesiology, Children’s Hospital of Nanjing Medical University, No. 72, Guangzhou Road, Gulou District Nanjing, 210008 People’s Republic of China

**Keywords:** Tracheal intubation anesthesia, OpenCV, Pierre-Robin syndrome

## Abstract

**Background:**

Difficult tracheal intubation is a common problem encountered by anesthesiologists in the clinic. This study was conducted to assess the difficulty of tracheal intubation in infants with Pierre Robin syndrome (PRS) by incorporating computed tomography (CT) to guide airway management for anesthesia.

**Methods:**

In this retrospective study, we analyzed case-level clinical data and CT images of 96 infants with PRS. First, a clinically experienced physician labeled CT images, after which the color space conversion, binarization, contour acquisition, and area calculation processing were performed on the annotated files. Finally, the correlation coefficient between the seven clinical factors and tracheal intubation difficulty, as well as the differences in each risk factor under tracheal intubation difficulty were calculated.

**Results:**

The absolute value of the correlation coefficient between the throat area and tracheal intubation difficulty was 0.54; the observed difference was statistically significant. Body surface area, weight, and gender also showed significant difference under tracheal intubation difficulty.

**Conclusions:**

There is a significant correlation between throat area and tracheal intubation difficulty in infants with PRS. Body surface area, weight and gender may have an impact on tracheal intubation difficulty in infants with PRS.

## Background

Difficult tracheal intubation is common in clinical practice, and it mostly refers to tracheal intubation that cannot be successfully completed by an ordinary indirect laryngoscope [1]. It represents the most difficult problem encountered by anesthesiologists in their daily work and is mainly caused by anatomical deformities, restricted back tilting activities, obesity and limited mouth opening [2]. These factors have an adverse effect on treatment. In practice, the level of difficulty is evaluated before the formal implementation of tracheal intubation. For patients with different levels of difficulty, preparations should be done in advance to avoid local mucosal damage caused by multiple intubation or complications such as dislocation of the circular cartilage [3].

In 2016, Münster et al [4] have reported that the position of vocal cords is related to laryngeal exposure and that difficult laryngoscopy is more likely to occur when vocal cords are closer to the head. From 2016 to 2018, many studies have utilized ultrasound for the clinical diagnosis of difficult tracheal intubation [5–10] Ultrasound provides not only real-time images but also reveals dynamic structural changes of the airway. In 2019, Lee et al [11] found that the distance from the mandibular groove to the hyoid bone and the distance from the inner edge of the mandible to the hyoid bone on X-ray images of the lateral neck were important for predicting difficult tracheal intubation in patients with acromegaly. However, there are only a few available methods for infant airway assessment and their accuracy is relatively poor [12].

Pierre Robin syndrome [13, 14] is the triad of micrognathia, glossoptosis, and cleft palate. These conditions could easily lead to difficult tracheal intubation which is the most significant risk factor for intubation anesthesia. Accurate preoperative prediction of intubation difficulty and adequate preparations are essential for ensuring successful airway management in infants with PRS. There are many methods for assessing the difficulty of tracheal intubation [3]; yet, no existing method is suitable for infants, especially infants with PRS. Moreover, few reports have focused on the application of CT on tracheal intubation difficulty assessment in infants with PRS [15, 16]. Therefore, this study was conducted to assess the difficulty of tracheal intubation in infants with PRS by incorporating CT to guide airway management for anesthesia [17].

## Methods

### Dataset

This retrospective study was approved by the Institutional Ethics Committee of Children’s Hospital of Nanjing Medical University and was conducted using the data obtained from Picture Archiving and Communication System (PACS) database and Operation Anesthesia Information System (OAIS) database. Informed patient consent was waived by our IEC. Clinical information and CT images were collected from infants with PRS who underwent intubation anesthesia in 2018 at Children’s Hospital of Nanjing Medical University.

Seven clinical risk factors [18] that may have an impact on tracheal intubation difficulty were provided by experienced clinicians, including gender, height, weight, body surface area (BSA), throat area, age, and pneumonia (Table 1). The calculation of the throat area was elaborated below, and the remaining indicators could be directly obtained or simply calculated. Tracheal intubation difficulty is divided into three levels based on whether glottis can be completely observed under visual laryngoscope, where level I refers to complete observation, level II refers to partial observation, and level III refers to the case when the only epiglottis can be observed.

**Table 1:** Clinical information for children with PRS

Gender	Male: Female	48: 48
Height (Unit: m)	Median (1st Qu., 3rd Qu)	0.5000 (0.5000, 0.5300)
Weight (Unit: kg)	Median (1st Qu., 3rd Qu)	3.400 (3.000, 3.800)
BSA (Unit: m^2^)	Median (1st Qu., 3rd Qu)	0.2190 (0.2050, 0.2330)
Throat area (Unit: pixel)	Median (1st Qu., 3rd Qu)	1440.5 (1237.2, 2034.4)
Age (Unit: day)	Median (1st Qu., 3rd Qu)	33.00 (13.67, 50.00)
Pneumonia	Yes: No	32: 64

Descriptive statistics of the seven clinical risk factors for 96 infants enrolled in the study. For categorical variables, the frequency of each category is listed. For numerical variables, the first quartile, median, and third quartiles are calculated

### Labeling criteria

**Fig. 1:**
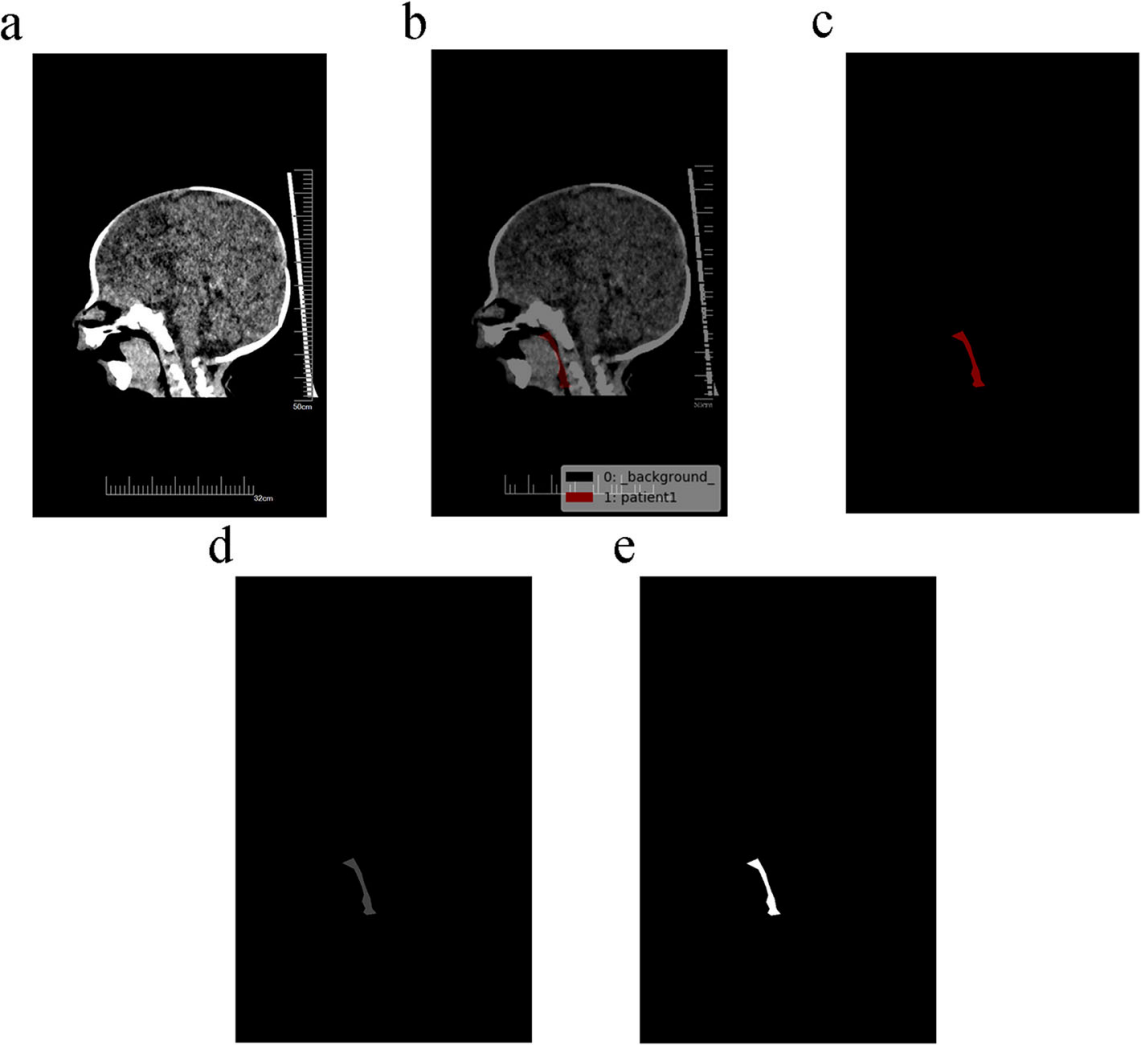
Images generated during area calculation. **a** Original CT image. **b** The image after labeling by labelme. **c** The *.png* image obtained by single-channel conversion. **d** The grayscale image obtained by color space conversion. **e** The binary image obtained after thresholding is performed

To assess the impact of the throat area on tracheal intubation difficulty, the collected CT images (Fig. 1a) were labeled according to the irregularity of the area being labeled using Labelme, an annotation tool which is based on the Python language and allows for irregular area annotation [19]. A radiologist with 20 years of clinical experience, who was blinded to the infants’ difficulty level, was responsible for labeling. Through a three-dimensional reconstruction technique, the median sagittal image of the upper airway of the infants was obtained, after which then the area of the oropharyngeal cavity (ie, the pharyngeal area between the plane of the tongue and the glottis) was labeled.

### Annotation file processing and area calculation

**Fig. 2:**
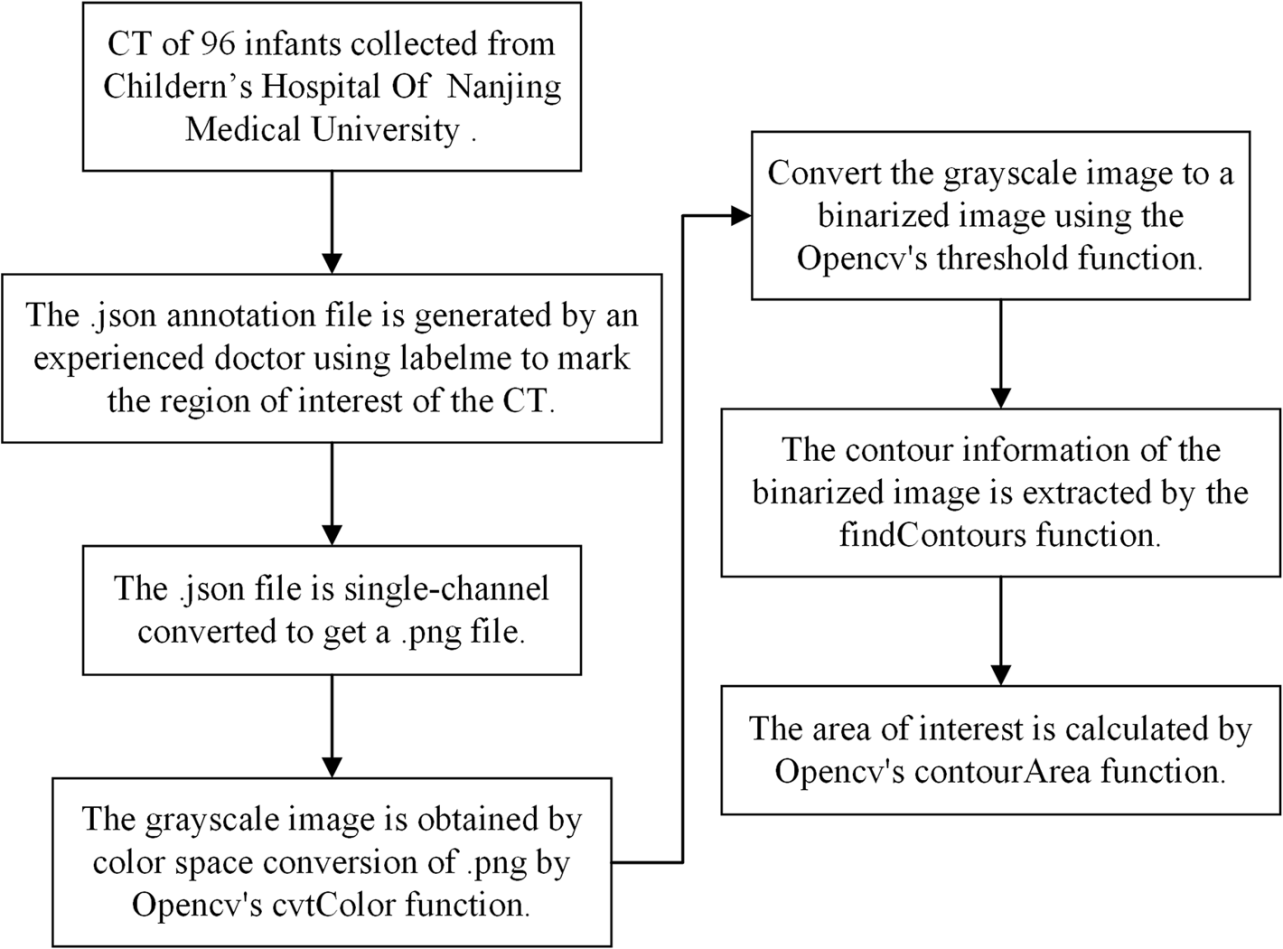
The flow chart for area calculation. The original image was processed by OpenCV for channel conversion, color space transformation, binarization, contour extraction and area calculation

The overall workflow is shown in Fig. 2. The annotation file generated by Labelme is in the format of .json (Fig. 1b) [20]. To calculate the throat area, the annotation file was first converted to a single-channel image in .png format (Fig. 1c).

OpenCV performed subsequent processing in the Python environment. First, the single-channel image that was obtained during the previous step underwent color space conversion using the cvtColor function of OpenCV and was converted into a grayscale image (Fig. 1d) [21, 22]. The grayscale image was then thresholded (the threshold was set to 1) using the threshold function and becoming a binary image (Fig. 1e) [22, 23]. The throat contour information of the marker was then obtained by the findContours function, with pixel position difference between two adjacent points in all contour points no larger than 1 [22, 24]. Finally, the contour information obtained in the previous step in the form of a point set was input into the contourArea function of OpenCV to calculate the area [22, 25].

### Correlation analysis

Correlation coefficients were used to assess the impact of each risk factor on tracheal intubation difficulty. Clinical risk factors highly correlated with difficulty level had better predicative effects in the clinic.

### Statistical analysis

Since clinical risk factors include numerical and categorical variables and tracheal intubation difficulty is categorical, the correlation was measured by the Spearman rank correlation coefficient. Besides, to analyze whether there is a significant difference in each clinical risk factor under tracheal intubation difficulty, the Kruskal-Wallis test was used for numerical factors, and Pearson’s Chi-squared test was used for categorical factors.

## Results

The flow chart of the study is shown in Fig. 2. Eight infants were excluded due to censored data (4 cases of censored pneumonia data and 4 cases of censored throat area data). Finally, 96 infants were included in the study, among whom 29 were level I difficulty, 43 were level II difficulty, and 24 were level III difficulty of tracheal intubation. Additional data with sufficient clinical information were collected.

**Fig. 3:**
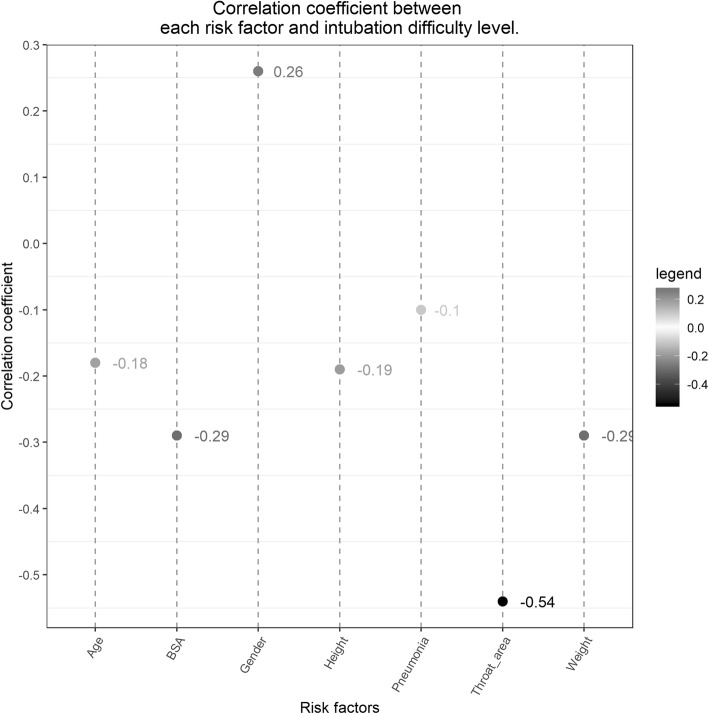
Correlation coefficient graph. The correlation between clinical risk factors and intubation difficulty level denoted by the Spearman rank correlation coefficient

The correlation coefficients are integrated in Fig. 3, where darker color indicates stronger correlations, while the lighter color represents weaker correlations. The correlation was strongest between the throat area and tracheal intubation difficulty with the correlation coefficient of − 0.54. Risk factors that were moderately correlated with tracheal intubation difficulty were BSA, weight, and gender with correlation coefficients of − 0.29, − 0.29 and 0.26, respectively. All numerical risk factors were negatively correlated with tracheal intubation difficulty. Among categorical risk factors, males were more difficult to intubate than females, and infants with pneumonia had a lower level of difficulty in intubation than infants without pneumonia.

The results of the internal difference analysis of risk factors are shown in Table 2. The difference in throat area under tracheal intubation difficulty was significant, with *P* < 0.0001 (Level I vs. II: *P* = 0.0022, Level II vs. III: *P* = 0.0002, Level I vs.III: P < 0.0001). The differences in BSA, weight, and gender under tracheal intubation difficulty were also significant, and their corresponding *P* values were 0.0117, 0.0117 and 0.0043, respectively. BSA, weight, and gender were significantly different when comparing level II to level III and level I to level III. Height, age, and pneumonia showed no significant difference under tracheal intubation difficulty.

**Table 2:** Difference analysis results of various factors

	Level 1 vs. 2	Level 2 vs. 3	Level 1 vs. 3	Total
Gender	1	0.0042^**^	0.0125^*^	0.0043^**^
Height	0.2473	0.4621	0.0526	0.1772
Weight	0.476	0.0264^*^	0.0025^**^	0.0117^*^
BSA	0.476	0.0264^*^	0.0025^**^	0.0117^*^
Throat area	0.0022^**^	0.0002^***^	< 0.0001^***^	< 0.0001^***^
Age	0.4694	0.2924	0.0503	0.1949
Pneumonia	0.4703	1	0.5253	0.5438

*P*-values for each risk factor under tracheal intubation difficulty. Among them, *P* values for a numerical variable were calculated by the Kruskal-Wallis test and for the categorical variable by Pearson’s Chi-squared test

*P < 0.05

**P < 0.01

***P < 0.001

## Discussion

In this study, we used clinical data from 96 PRS infants who underwent intubation anesthesia to perform correlation analysis, which demonstrated that the throat area had a significant effect on tracheal intubation difficulty. Our results revealed that a larger throat area was associated with a lower level of tracheal intubation difficulty, which is consistent with the clinician’s subjective perception. Besides, we found that high BSA and weight corresponded to low tracheal intubation difficulty, which may be related to the better physical development of these infants. Moreover, male infants had a higher tracheal intubation difficulty than females. Pneumonia, age, and height were slightly correlated with the difficulty of tracheal intubation, which may be due to the small amount of collected data and thus needs to be further analyzed.

After further *P*-value analysis, we found that four factors, namely throat area, gender, weight, and BSA, were internally different under the difficulty of tracheal intubation. Among them, the difference in the throat area was significant between all levels of tracheal intubation difficulty. Gender, weight, and BSA were only significantly different between level II and level III, level I, and level III. We speculate that it may be because the sample size of the level I tracheal intubation difficulty is too small. In addition height, age, and pneumonia under tracheal intubation difficulty were not statistically significant, which may be related to the small sample size.

Attention should be paid to some of the limitations of our research. First, we studied the correlation between risk factors and tracheal intubation difficulty without building a predictive model, because the limited number of cases obtained in this study could not meet the requirements for modelling. Second, in order to facilitate the drawing of the correlation coefficient map, the correlation measure was based on the Spearman rank correlation coefficient. In addition, this was a single-center study. Finally, the annotation of the region of interest in the throat was done by one experienced doctor, which may be subjectively biased.

This study has few limitations: first, future studies should expand the number of cases collected and construct a predictive model of intubation difficulty. Secondly, the regional annotation should be performed by multiple physicians, and artificial intelligence annotation tools should be constructed. Finally, the integration of labeling and difficulty prediction should be performed.

## Conclusion

The throat area may be helpful for predicting the difficulty of tracheal intubation in infants with PRS. Besides, gender, weight and BSA may also affect the prediction of the difficulty of airway intubation to some extent.

## Data Availability

The datasets used and/or analyzed during the current study are available from the corresponding author on reasonable request.

## Abbreviations

PRS: Pierre Robin Syndrome
CT: Computed Tomography
BSA: Body Surface Area
